# Folie a Deux: Shared Psychotic Disorder in a Medical Unit

**DOI:** 10.1155/2021/5520101

**Published:** 2021-09-25

**Authors:** Saumya Bhutani, Damir Huremovic

**Affiliations:** ^1^Zucker Hillside Hospital at Northwell Health, 75-59 263rd Street, Glen Oaks, NY 11004, USA; ^2^Donald and Barbara Zucker School of Medicine at Hofstra/Northwell, Hempstead, NY 11549, USA; ^3^North Shore University Hospital, 300 Community Drive, Manhasset, NY 11030, USA

## Abstract

**Introduction:**

A shared psychotic disorder is a system of delusions shared by two or more individuals. Shared psychotic disorders typically develop in pairs or groups with a close relationship who are socially isolated. The function and affect of those inflicted with shared psychotic disorders usually remain intact. For these reasons, a shared psychotic disorder is seldom identified, diagnosed, and treated. This case describes a shared psychotic disorder incidentally discovered in a medical unit.

**Case:**

The patient was a 47-year-old woman with no known past psychiatric history who had been medically admitted for gastroenteritis. On the day of discharge, a psychiatric consult was requested for “paranoia and bizarre behavior.” The patient was seen making statements that she needed security and the FBI to escort her as she left the hospital. Another person in the patient's room was discovered to be the patient's mother who had been staying with her in the hospital. Evaluation of the patient along with observation of her mother revealed that the two shared a complex system of delusions revealing a diagnosis of shared psychotic disorder. *Discussion*. A shared psychotic disorder is a unique psychiatric diagnosis. It may be even rarer to diagnose in the inpatient medical setting because multiple individuals from a shared system are typically not seen. In this case, the patient and her mother had multiple clinical characteristics of a shared psychotic disorder, including an enmeshed relationship and social isolation. The treatment for shared psychotic disorders involves separation of the individuals and pharmacotherapy with antipsychotics. This case also presented a unique ethical dilemma as the psychiatric team was called to evaluate a patient and found a patient and another individual to have symptoms.

**Conclusion:**

A shared psychotic disorder is important to consider on the differential when cases of psychosis with delusional systems are seen on medical floors.

## 1. Introduction

A shared psychotic disorder refers to delusions that are shared between two or more people. The concept of delusional ideas being shared by two or more individuals was first documented in France in the 19^th^ century [1]. The term *folie à deux* was coined in 1877, translating literally to “madness for two” and referring to delusions shared amongst two individuals [2]. While shared psychotic disorders are not limited to two individuals, the *deux* is replaced with however many people are within the system of beliefs. *Folie à deux* thus represents the simplest and most frequent variant of shared psychotic disorders. When Laségue and Falret first introduced the term *folie à deux*, they posited three criteria that were part of the condition as follows: within a pair, a more active and intelligent individual imposes delusions onto a more passive, less intelligent individual; both individuals suffering from the delusions live together closely without any external influence; and the delusions have some degree of likelihood referencing “common experience, hopes, and anxieties” [1, 2].

Since the initial descriptions of *folie à deux*, multiple clinical subtypes have been described in the literature with Gralnick outlining four in his seminal review in 1942 [3]. Laségue and Falret's description was dubbed “imposed psychosis”; in this subtype, the passive “recipient” does not resist the delusions from the more active “inducer” yet the “recipient” will abandon the delusions if separated from the “inducer” [2, 4]. In communicated psychosis, the “recipient” only accepts the delusions from the “inducer” after much resistance; however, once the “recipient” accepts them, they adopt them long-term, even if separation between the two individuals occurs [5]. Simultaneous psychosis occurs when delusions appear at the same time in both individuals, usually with a trigger [6]. Finally, in induced psychosis, an individual induces delusions in a “recipient” who already had other preexisting delusions, suggesting some susceptibility to developing delusions [4].

Throughout the years, additional terms to describe these phenomena were introduced, including “psychosis of association,” “double insanity,” “induced psychosis,” and “imposed psychosis” [7]. The central tenet in this disorder, regardless of the evolution of the definitions and subtypes, is the existence of delusional ideas amongst multiple people. The DSM-V focuses on this broad concept, while other aforementioned differentiations have little clinical significance today.

Shared psychotic disorder, or *folie à deux*, is listed in DSM-V under the “Other Specified Schizophrenia Spectrum and Other Psychotic Disorder” category as “delusional symptoms in the partner of an individual with delusional disorder.” It defines that “in the context of a relationship, the delusional material from the dominant partner provides content for delusional belief by the individual who may not otherwise entirely meet criteria for delusional disorder” [8].

Some common themes and risk factors that have emerged in the study of *folie à deux* reflect the historical descriptions. These include a close, prolonged relationship between the inducer of the delusion and the recipient, a lack of another psychotic disorder explaining the development of the delusions, a delusion that is usually persecutory in nature, and maintenance of function without intellectual or affective disorganization in those afflicted [7]. The main risk factor for the development of *folie à deux* is extreme social isolation [9]. Other risk factors for the “recipient” include a passive personality, cognitive impairment, language difficulties, and negative life events [10]. These themes, particularly the close relationship between those sharing the delusion, their social isolation, and the maintenance of their function, are likely responsible for the underestimation of incidence of shared psychotic disorders.

Patients with shared psychotic disorders are seldom identified as such, often do not meet criteria for involuntary psychiatric hospitalization, and go back to the environment that supports and reinforces the psychosis [7]. It is exceptionally rare to see a shared psychotic disorder in medically hospitalized patients, and even then, it may be an incidental finding as in the case described here. Patients on medical floors are almost invariably seen as individuals and rarely within the familial context; hence, shared psychotic disorders seldom get consideration in the differential diagnosis.

## 2. Case Report

The patient was a 47-year-old woman with no known past psychiatric history and past medical history of chronic back pain who had been medically admitted for uncomplicated gastroenteritis. Her weeklong hospital course was unremarkable until the day of discharge, when a psychiatric consult was requested for “paranoia and bizarre behavior.”

As per the medical team, upon discussing discharge, the patient began behaving oddly. She stated that she felt unsafe and demanded that the staff call the FBI to investigate. Earlier the patient was seen hiding underneath her hospital bed. The patient told staff she could not leave the hospital unless security has “secured the perimeter.”

On initial encounter, the patient was found attempting to use the phone by the nursing station to call the “authorities.” She resisted engaging in a psychiatric interview, stating, “I don't need psychiatry, I need legal services because a crime was committed.” With difficulty, she was redirected back to her room where another older female person, dressed in a hospital gown, and presumed to be another patient, was seated beside the second bed. In the midst of the team's attempts to elicit the patient's concerns, that other older woman spontaneously stood up and addressed the team: “She is telling the truth. You have to believe her! Our lives are in jeopardy and we're just going to be labeled as ‘crazy' by these psychiatrists.” The other woman insisted that the psychiatric interview with the patient could not be conducted in the patient room because it was “tapped and wired.”

The team stepped outside to inquire with the staff about the other patient and learned that the other “patient” was actually the patient's mother, who had been allowed to stay overnight with the patient for the past several days. The patient and her mother were then carefully separated, and the patient was interviewed first.

Throughout the interview, the patient was guarded. She explained that she was admitted to the hospital for vomiting and diarrhea and treated with antibiotics. The patient maintained that she should not be talking to psychiatry but rather to the police because an unspecified “crime was committed.” She described the crime as “the worst form of verbal abuse” and denied any physical or sexual assault. She was unable to provide adequate answers on why she had not yet reported the crime or why things had exacerbated now. She was also unable to elaborate if and how her safety and the safety of her mother would be jeopardized. The patient stated she would like security to escort her out of the hospital. She shared how she was thinking about hiring a lawyer and a personal bodyguard. When asked about the events of the morning, the patient stated that she and her mother got into a dispute after she confided to her mother the true extent of the “crime.”

The patient's labs had been completed earlier in her hospital course prior to this evaluation. She had been noted to be anemic with no symptoms. A hematology consult and work-up were done and found low iron and a slightly low vitamin B12 with no acute interventions necessary. A thyroid stimulating hormone was not completed. A comprehensive metabolic panel and urinalysis were within normal limits. Stool and blood samples were negative for growth of bacteria. A computed tomography (CT) scan of the abdomen and pelvis was unremarkable. A head CT was not done prior to this clinical encounter as the patient had not demonstrated any altered mental status or bizarre behavior. Throughout the hospitalization, the patient was treated with intravenous fluids, antibiotics azithromycin and metronidazole, acetaminophen for pain, and ondansetron for nausea. None of these medications were suspected to be responsible for her presentation.

The patient denied any past psychiatric history as well as past medical history except for chronic back pain. Review of the electronic medical record also did not reveal any additional past psychiatric or past medical history. The patient stated she and her mother lived together alone since the death of her father 27 years ago. The patient did not work; she received income via disability for her chronic back pain. The patient denied any substance use. Family history was notable for Cushing's disease and asthma in her mother and asthma and diabetes in her father.

The patient consented for the treatment team to speak with her mother with the patient present. During the joint interview, the patient's mother was fearful and anxious. She repeatedly stated that she thought it was not safe for the two of them to return to their home. The mother attempted to share more details behind their delusions, but the patient kept interrupting and preventing her from sharing more. The patient interjected, “No, these are not people that are going to help us. I need to be discharged and seek legal services and the police.” The patient's mother began to make gestures towards the treatment team. She stated, “Can't you take a hint? Do you not understand English?” She pleaded, “Don't you see? We're scared! We need help!” The mother yelled, “We can't say anything more!” She then mouthed the words, “We're wired. We're tapped. They're listening!” and made a motion with her hand suggesting her throat will be slit if she says anything else. At this point, the mother appeared more preoccupied with and terrified by the content of their mutual suspicions and concerns.

The patient gradually subsided in her agitation and began to make a case to the team and to her mother that they can both go home and manage everything later with a lawyer. Her mother challenged her, and the two of them argued back and forth for a while, with the treatment team, staff, social worker, and nursing administrators attempting to understand the situation and obtain more information. They did not have any other sources of collateral that could have been contacted.

As she became calmer, the patient's reality testing improved. She stated that she felt safe returning home without security escort. The patient gained enough insight to understand that she was getting unwanted psychiatric attention and was able to change her presentation in order to get herself discharged once she realized the requests she made for increased security were not going to be granted. Less distressed and more able to engage with the team, she accepted the possibility of seeking mental help. The patient was offered a one-week supply of quetiapine 50 mg nightly although it was unclear if she would actually take it and actually follow-up at the hospital's outpatient walk-in crisis mental health clinic. However, despite a concern that the patient may not follow-up, her overall risk of imminent harm was still not high enough to mandate treatment through involuntary hospitalization. The patient did not meet criteria for involuntary inpatient psychiatric hospitalization and declined a voluntary one. She accepted resources for outpatient treatment for both her and her mother. She understood that she should call 911 or go to the nearest emergency room should she feel that she or her mother were in imminent danger. The patient told her mother that should her mother feel unsafe, she would find them another place to stay.

The patient's mother, who now appeared more distressed and disturbed, was not the patient at the hospital and would require a transfer and an emergency department (ED) registration, in order to receive a formal evaluation. After careful consideration, taking into account the mother's age and the patient's ability to act as the responsible caretaker, which became more and more evident towards the end of the encounter, the team decided against committing the patient's mother to an ED evaluation.

## 3. Discussion

Shared psychotic disorder, or *folie à deux*, is a unique psychiatric diagnosis in which delusional ideas are shared amongst two or more people. Because of the rarity in identifying these cases, much of what is known about the disorder comes from case reports and case reviews [11].

This case features a medically hospitalized patient who exhibits paranoia and bizarre behavior. There were no medical etiologies or substances that explained the patient's presentation. The patient and her mother shared an intricate series of delusions. Based on their demeanor and confirmed through a subsequent recent clinical encounter with the patient since this first one, the patient and her mother's delusions had been going on for some time with ebbs and flows in course. In this case, there were low concerns for either of the individuals' malingering or gaining to seek a sick role as in a factitious disorder. They both were very emotionally distressed, and this had been a distinct, dramatic departure from how the patient presented for medical hospitalization. Neither of them was known to have prior psychiatric treatment or another psychiatric diagnosis explaining their symptoms. Their shared delusions had likely been transferred from one to the other, and their leading diagnosis was a shared psychotic disorder, as defined by DSM-V.

The patient and her mother also meet the characteristics of *folie à deux* described in the literature historically. Returning to Laségue and Falret's criteria, the pair appears to have lived alone together for many years with an enmeshed relationship. Neither was employed, and they were unable to offer sources of collateral; thus, it is assumed that they were free of external influence and were at high risk of developing shared psychosis due to their social isolation.

Based on the clinical interview, the pair's delusions appear to be persecutory in nature, and case reviews have found that persecution is the most common delusional theme in *folie à deux* [10, 12]. The original definition of *folie à deux* determined that the delusions had some degree of likelihood “for it to be communicable” [2, 7]. The patient and her mother provided limited details about their delusions, but crime and wiretapping were perhaps more likely than the delusions seen in psychotic disorders such as schizophrenia, including thought broadcasting, insertion, and withdrawal, Capgras delusions, or Truman delusions.

As previously mentioned, the extent and intensity of their symptoms had not elicited psychiatric care for the patient and her mother prior to this encounter. The patient even spent the majority of her hospitalization without causing any alarm for the medical team to believe that a psychiatric consultation was warranted. Thus, it is likely that, while suffering from these delusions, their functioning was at least marginally preserved without disorganization of their routines. This is commonly seen in *folie à deux*, reinforcing this diagnosis as opposed to another in which functioning and organization are more impaired, and lends itself to the difficulty of recognizing and learning more about this disorder [7]. Epidemiologically, in more than 90% of cases, the individuals experiencing a shared psychotic disorder belong to the same family with a high frequency of parent-child pairs, as seen here [7, 10–12]. In these multiple ways, the patient and her mother met the clinical characteristics of *folie à deux*.

Due to the limited nature of this clinical encounter, it was difficult to determine if this case fit into the subtypes of *folie à deux* described in the literature. We were also unable to determine who, the patient or her mother, was the “recipient” and who was the “inducer” of the delusions. Studies have found that there is no difference between sex and age between the “inducer” and “recipient,” so it cannot be assumed that the mother, older, would be the “inducer” [7]. Reflecting on the clinical interview, control and dominance as well as delusional content between the patient and her mother shifted. The patient initially shared that the acute exacerbation of her symptoms was triggered by a conversation in which she informed her mother more information about the “crime.” This may suggest that the patient has been the “inducer” of the delusions as she was sharing more about them, but it is possible that she was actually the “recipient” who has elaborated on the delusions, which did occur [7]. While the pair was arguing about how much information to disclose to the psychiatric consultation team as well as the hospital staff, the patient ultimately assumed the more dominant role and determined the course of the conversation. Again, we may speculate, in this context, that the mother was the more passive and submissive, which puts her at risk of being the “recipient.”

Research about the treatment of *folie à deux* suggests that there are two parts: separation of those afflicted and pharmacotherapy with antipsychotics [1]. In this case, the consult-liaison psychiatric team's primary decision, after evaluating this patient, who was now medically cleared, was determining further disposition. The options included offering treatment through community referrals or voluntary hospitalization or mandating treatment through involuntary hospitalization. Hospitalization of the patient would have likely enabled separation of the pair and led to an initiation of treatment with antipsychotics. A short course of low-dose quetiapine was offered to the patient at discharge. Antipsychotics are thought to treat shared psychotic disorders by resolving the delusions; however, additional antidepressants and anxiolytics may help with secondary depression and anxiety. This low dose of quetiapine would likely not affect any psychotic symptoms or delusions but more likely help with associated anxiety and insomnia.

This case presents a unique position that psychiatrists may be in when observing *folie à deux* in a medical setting. We were asked to evaluate a patient and found a patient and relative that may both benefit from psychiatric care. Collateral, which we often use to aid our treatment decisions, was limited due to the nature of this illness—the only collateral the patient had was her mother who exhibited similar symptoms.

Ultimately, we found it was only ethical for us to evaluate the patient and make treatment recommendations for her and not her mother. Had we felt that either of them was posing a threat to their safety, this outcome may have differed leading to inpatient admission for the patient or referral to the emergency room for her mother. Based on the safety assessment completed, the patient, who was medically cleared, was safe to be discharged home with resources on outpatient treatment.

In a primary medical hospital setting, the opportunity to see multiple individuals from a shared system is rare. The most likely scenario of shared psychotic disorders on medical floors is in shared delusional parasitosis [13, 14]. Thus, shared psychotic disorders may be more common on medical floors than is known, further contributing to its underestimated incidence.

A shared psychotic disorder is a psychiatric disorder in which more than one person has the same series of delusions. Case reports and reviews in the literature have elicited numerous definitions, clinical characteristics, and risk factors. Because those afflicted tend to remain together in close contact with isolation and preservation of function, psychiatrists are rarely faced with this presentation. When they are, it may be incidental. The treatment of *folie à deux* is also challenging because the people afflicted reinforce the delusions and, thus, are less likely to seek or be referred to care. Shared psychotic disorders are important to consider on the differential when cases of psychosis with delusional systems are seen on medical floors.
